# MAGE: An Open-Source Tool for Meta-Analysis of Gene Expression Studies

**DOI:** 10.3390/biology11060895

**Published:** 2022-06-10

**Authors:** Ioannis A. Tamposis, Georgios A. Manios, Theodosia Charitou, Konstantina E. Vennou, Panagiota I. Kontou, Pantelis G. Bagos

**Affiliations:** 1 Department of Computer Science and Biomedical Informatics, University of Thessaly, 35131 Lamia, Greece; itamposis@uth.gr (I.A.T.); gmanios@uth.gr (G.A.M.); tcharitou@uth.gr (T.C.); kvennou@uth.gr (K.E.V.); 2 Department of Mathematics, University of Thessaly, 35131 Lamia, Greece; pkontou@uth.gr

**Keywords:** meta-analysis, gene expression studies, multiple outcomes, differentially expressed genes, enrichment analysis

## Abstract

**Simple Summary:**

In this work we present MAGE, an open-source Python package developed for the meta-analysis of gene expression data. It contains functions to convert probes to gene identifiers, and to perform standard meta-analysis, meta-analysis with bootstrap standard errors, and meta-analysis of multiple outcomes, as well as functional enrichment analysis. Additionally, visualizations for every function of this software package are provided. MAGE is available both in a standalone version and as a webserver.

**Abstract:**

MAGE (Meta-Analysis of Gene Expression) is a Python open-source software package designed to perform meta-analysis and functional enrichment analysis of gene expression data. We incorporate standard methods for the meta-analysis of gene expression studies, bootstrap standard errors, corrections for multiple testing, and meta-analysis of multiple outcomes. Importantly, the MAGE toolkit includes additional features for the conversion of probes to gene identifiers, and for conducting functional enrichment analysis, with annotated results, of statistically significant enriched terms in several formats. Along with the tool itself, a web-based infrastructure was also developed to support the features of this package.

## 1. Introduction

High-throughput techniques, such as microarrays or RNAseq, are widely used to assess simultaneously the expression of thousands of genes under certain conditions, and to study the effects of treatments, diseases, or developmental stages. Single experiments cannot capture the bigger picture, and small studies are not easily replicated. Meta-analysis is a valuable tool for the synthesis of evidence across studies [1]. Several programs for the meta-analysis of gene expression data are also available, such as metaMA [2], MetaIntegrator [3], DExMA [4], MetaDE [5], and Network Analyst’s meta-analysis module (Express Analyst) [6]. These tools (except for Network Analyst, which is a webserver) are standalone tools. However, these tools have some limitations, since they cannot handle multiple outcome data, they do not provide a gene enrichment analysis, and they all use only the standard methodology for meta-analysis. Functional enrichment analysis is commonly performed to measure the significance of functional enriched terms (GO, KEGG, reactome, protein domains, diseases) in a set of candidate genes, which usually emerge as the final outcome of a meta-analysis. A hypergeometric test evaluates the over-representation of candidate genes compared to a set background list of genes and identifies the statistically significant enriched terms. There are several methods used for functional enrichment analysis, which vary across different tools, such as g:Profiler [7], aGOtool [8], WebGestalt [9], PANTHER [10], and DAVID [11], by integrating different annotation databases, computing algorithms, and statistical methods. 

In this article, we present MAGE, an open-source tool which is specifically designed for the meta-analysis of gene expression studies. The purpose of this work is to present an easy-to-use software package that will facilitate the meta-analysis of gene expression studies, offering several functionalities that are not available in other tools. The tool is offered as both a Python package and as a freely available web server, and offers the options of standard methods for meta-analysis (random effects model), bootstrap standard-errors, corrections for multiple testing, and multiple outcomes meta-analysis, as well as tools to convert datasets from different platforms, in which case the probe identifiers are converted to gene identifiers.

## 2. Materials and Methods

MAGE can be used to perform a broad range of tasks related to the meta-analysis of gene expression data. It consists of four components:

1. Annotation and uploading of gene expression study files;
2. Conversion of probes to gene identifiers;
3. Performing meta-analysis with various methods;
4. Conducting functional enrichment analysis.

**Figure 1:**
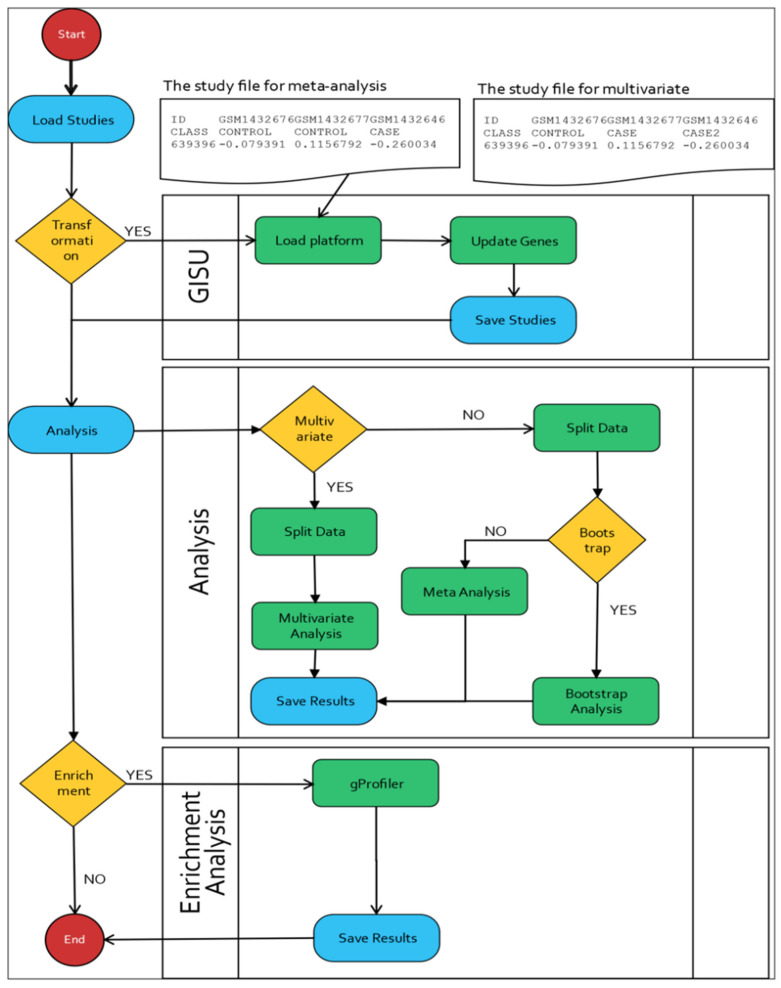
Schematic representation of the workflow.

A brief description of these components is provided in the following sections. In order to summarize MAGE’s functionality, we briefly show its features in a schematic representation of the workflow in Figure 1. It is important to keep in mind that guidelines described in tutorials and methodology papers for the meta-analysis of microarray studies [12,13] suggest that the user has performed all of the necessary pre-processing and normalization steps prior to performing the meta-analysis. As such, we advise users to employ the methods they deem appropriate prior to pooling the datasets. MAGE was primarily designed for microarray studies; however, under some circumstances, the same methodology can be applied to RNAseq data. Of course, in this case, pre-processing and normalization are also needed.

### 2.1. The Upload and Annotation Step

**Figure 2:**
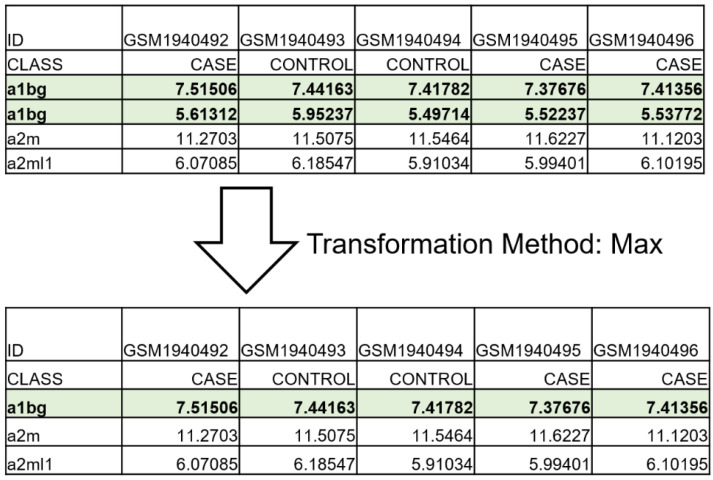
An example of how GISU transforms probes to gene identifiers. Two probe identifiers correspond to the same gene identifier (highlighted gene identifier). In this case, the max transformation method is applied. For each subject, the largest values remain in the final dataset, and the other is deleted.

In the first step, each study file should be uploaded in tab-delimited format (.txt) as shown in the Figure 2. The first row should be named ID, and the next column should contain the experiment subjects’ names (e.g., GSMxxx). The second row should be named CLASS, and the next columns contain subjects’ status (for an analysis with a single outcome, the CONTROL and the CASE were used to indicate the status of the controls and the patients in the study). The subsequent lines should have either the gene symbol identifiers of the experiment, or the platform’s probe identifiers (if the GISU module is to be used) and the expression value of each probe per subject.

### 2.2. GISU Component

MAGE uses an optional component called GISU (gene ID/symbol update) to transform the platform’s probe identifiers to gene symbols identifiers. This can be helpful when one is comparing datasets arising from different platforms, in which case the probe identifiers must be converted to gene identifiers. Considering that multiple probes may correspond to the same gene in a microarray experiment [14], the multiple entries of the same gene can be combined, resolving the “many-to-many” relationship between probes and gene symbols. The software offers three options for this task: the minimum, maximum, or arithmetic mean (average) [12,14]. If a particular platform is not included in the list, the user can upload the platform file in order to proceed to the transformation.

### 2.3. Standard Meta-Analysis

In a standard approach for random effects meta-analysis [15], we use as effect size the sample estimate of the standardized mean difference, known as Cohen’s *d*. The exact formula of Cohen’s *d* is given by:

(1) $$d_{i}=\frac{{\bar{X}}_{1_{i}}-{\bar{X}}_{2_{i}}}{Sp_{i}}$$

and the pooled standard deviation Sp is given by:

(2) $$Sp_{i}=\sqrt{\frac{\left({n_{1}}_{i}-1\right){{S_{1}}_{i}}^{2}+\left({n_{2}}_{i}-1\right){{S_{2}}_{i}}^{2}}{{n_{1}}_{i}+{n_{2}}_{i}-2}}$$

where ${\bar{X}}_{1_{i}}$, ${\bar{X}}_{2_{i}}$ are the means of the expression of the control group and cases group, respectively, in the *ith* study, ${S_{1}}_{i}$, ${S_{2}}_{i}$, are the standard deviations of the two groups, and ${n_{1}}_{i},\;{n_{2}}_{i}$ are the sample sizes of the two groups.

d, usually overestimates the absolute value in small samples, something that can be corrected using the so-called Hedge’s g, which generates an unbiased estimate using a correction factor, *J*. We use the exact formula for *J* given in [16], following which, the Hedge’s g correction is applied on Cohen’s d: $g_{i}=Jd_{i}$ with *J* given by:

(3) $$J\left(v_{i}\right)=\frac{\Gamma \left(\frac{v_{i}}{2}\right)}{\sqrt{\frac{v_{i}}{2}}\;\Gamma \left(\frac{v_{i-1\;}}{2}\right)}$$

where $v_{i}=n_{1_{i}}+n_{2_{i}}-2$.

In the meta-analysis of gene expression data, two measures are routinely used to quantify the overall findings of the meta-analysis. IDR (integration-driven discovery rate) [17] and IRR (integration-driven revision rate) [18] denote the percent of differentially expressed genes in the meta-analysis that are not differentially expressed in any of the individual gene expression studies, and the percent of genes that were not identified as differentially expressed in the meta-analysis but differentially expressed in at least one individual gene expression study, respectively. IDR and IRR are given by:

(4) $$IDR=\frac{\#genes\left[\;p_{i}\;\leq \;x\;in\;meta-analysis\;\right]\;and\;[\;p_{i}>x\;\;in\;individual\;studies]\;}{\#genes\left[\;p_{i}\;\leq \;x\;in\;meta-analysis\;\right]}$$

(5) $$IRR=\frac{\#genes\left[p_{i}\;\leq \;x\;\;in\;at\;least\;one\;\;study\;\right]\;and\;[\;p_{i}>x\;in\;meta-analysis]\;}{\#genes\left[\;p_{i}\;\leq \;x\;\;in\;at\;least\;one\;individual\;study\;\right]}$$

where *p_i_* is the *p*-value obtained by the statistical test of differential expression for the *ith* gene, and *x* is the desired threshold of statistical significance or FDR used to assign differential expression.

### 2.4. Bootstrap Standard Errors

Gene expression experiments often suffer from the problem of very small sample size, which may result in problems in the estimation of statistical significance. Although the Hedges *g* correction improves the situation, some further improvements are needed. Bootstrap is a statistical method for estimating the sampling distribution of an estimator by sampling with replacement from the original sample [19]. The bootstrap method has been applied in microarray experiments, and empirical evidence suggests that it produces accurate estimates, at least for moderately small sample sizes (~10 individuals). This feature, being computationally demanding, is only available in the standalone version, where the user may choose the number of repetitions. It is suggested that users run this function with more than 200 repetitions, to achieve accurate estimations [20].

### 2.5. Multiple Outcomes Meta-Analysis

There are situations in which we have a comparison of more than two groups. In general, we may encounter multiple outcomes, multiple risk factors, or multiple treatments. In such situations, we have three groups instead, and subsequently two effect sizes (*g*_1*i*_, *g*_2*i*_) that are calculated for the comparison of two groups or conditions against a reference category. In all cases, we are usually interested in finding differentially expressed genes, common in all conditions, or genes that differ among the conditions. The multivariate meta-analysis provides several important advantages and there are available methods to handle this situation [21]. However, these methods come with a computational cost and, thus, in order to enable faster analysis, we followed a different approach: within each study we first performed either the tests (described in [21]) for the equality of the estimates ($D_{i}=g_{1_{i}}-g_{2_{i}}=0$), or the joint test with the null hypothesis stating that both estimates are equal to zero ($W_{i}$), and we then combined these tests across studies with standard methods of univariate meta-analysis. $W_{i}$ is given by:

(6) $$W_{i}={\left[\begin{array}{c} {\hat{g}}_{1_{i}}\\ {\hat{g}}_{2_{i}}\; \end{array}\right]}^{T}{\left[\begin{array}{cc} v\hat{a}r\left({\hat{g}}_{1_{i}}\right) & c\hat{o}v\left({\hat{g}}_{1_{i}},{\hat{g}}_{2_{i}}\right)\\ c\hat{o}v\left({\hat{g}}_{1_{i}},{\hat{g}}_{2_{i}}\right) & v\hat{a}r\left({\hat{g}}_{2_{i}}\right) \end{array}\right]}^{-1}\left[\begin{array}{c} {\hat{g}}_{1_{i}}\\ {\hat{g}}_{2_{i}} \end{array}\right]$$

### 2.6. Multiple-Comparison Methods

A well-known problem in high-throughput experiments is the need to adjust for multiple comparisons, since in such cases the nominal level of significance is not preserved. Following this rationale, MAGE provides methods that correct for multiple-comparison correction. We implement several methods that control the family-wise error rate (FWER), and methods that control the false discovery rate (FDR). For the family-wise error rate, the Bonferroni, Holm, Holand, and Sidak methods were implemented [22,23,24], whereas for the False Discovery Rate, we implemented the Hochberg and Simes correction methods [25,26]. To consider a gene as statistically significant with these correction methods, the standard *p*-value of a gene needs to be smaller than the *p*-value coming from the selected correction method ($p_{i}<p_{cor_{i}}$).

The Bonferroni correction is given by: $p_{cor_{i}}=a/n$ and the Sidak method is given by: $p_{cor_{i}}=1-{\left(1-a\right)}^{1/n}$, where a is the selected level of significance and n is the total number of tests. For the Holm and Holland methods, the list of *p*-values needs to be sorted in ascending order, and the *p*-values are corrected with the following formulae: $p_{cor_{i}}=\frac{a}{n+1-i\;}$, $p_{cor_{i}}=1-{\left(1-a\right)}^{n+1-i}$, respectively.

To correct the *p*-values with the Hochberg and Simes methods, the list of *p*-values needs to be sorted in ascending order, but the comparisons are carried out in reverse order, i=n, n−1,…1. The Hochberg correction is given by: $p_{cor_{i}}=\frac{a}{n+1-i}$, which is similar to the Holm method, and the Simes correction method is given by $p_{cor_{i}}=a\frac{i}{n}$.

### 2.7. Enrichment Analysis

Finally, the software uses g: Profiler [7] to perform functional enrichment analysis with a given gene list, produced as the result of the meta-analysis, by using the implemented Python module. The software returns several files containing gene definitions, a list with statistically significant enriched GO terms, biological pathways, regulatory motifs in DNA, or phenotype ontologies with which these genes are highly enriched, and provides the user with the option to visualize results with a Manhattan or a heatmap plot.

### 2.8. Implementation

The MAGE toolkit is implemented in Python and as a freely available web server, using an interface of PHP as well as CSS and JavaScript. It is available as a web service, and as a standalone package through a GitHub (https://github.com/pbagos/mage) repository under the GNU license. The online version of MAGE can be found on the website of compgen.org (http://www.compgen.org/tools/mage, http://195.251.108.230/MAGE/), where the user can perform its functions by selecting the analysis options easily and interactively. All functions of the standalone tool that are described above can be run in the online tool, except the Bootstrap meta-analysis function, as it is time consuming and requires many repetitions in order to give accurate estimations. 

### 2.9. Plots

**Figure 3:**
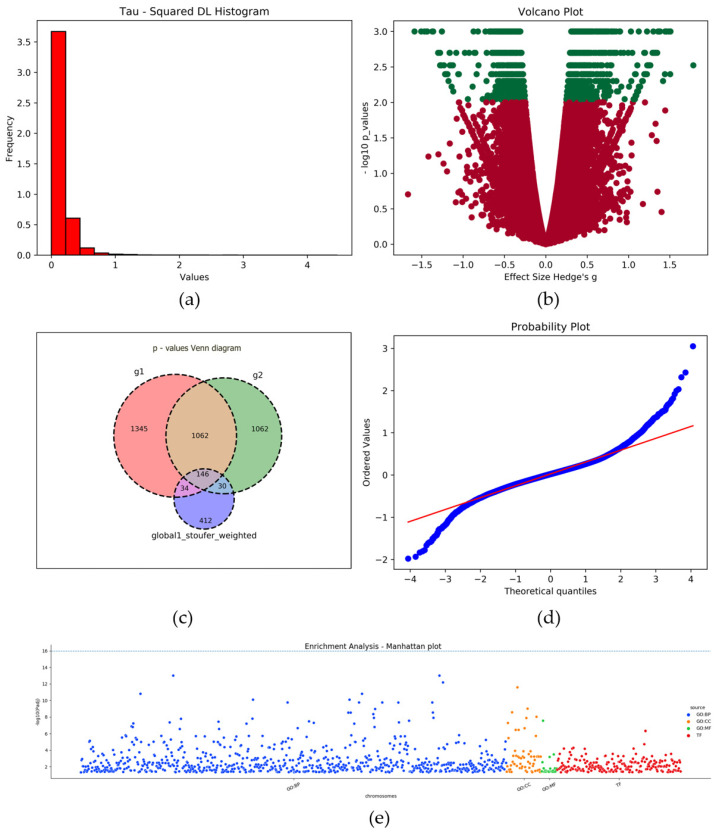
Plots generated by MAGE. (**a**) Tau-squared histogram; (**b**) volcano plot; (**c**) Venn diagram; (**d**) Q–Q plot; (**e**) Manhattan plot. Similar histograms such as (**a**) are produced for the other heterogeneity measures. The heterogeneity measure histograms, i.e., (**a**) the Volcano plot (**b**) and the QQ plot (**d**), can be produced both from the standard meta-analysis and the bootstrap meta-analysis functions. The three circle Venn diagram (**c**) is implemented for the multiple outcomes meta-analysis, and the Manhattan plot (**e**) occurs from the functional enrichment analysis. A full list with the enriched GO term table is provided in the enrichment analysis results file that is given in Supplementary File S1.

Graphical representations of the results are of great importance, so several standard graphical outputs, such as Q–Q plots, volcano plots, Manhattan plots, and heatmap plots, have been implemented for each significantly enriched GO term. With the Q–Q plot, a theoretical distribution of the corrected effect sizes is compared to the actual distribution of the corrected effect sizes. In addition, histograms were used to portray the measures of heterogeneity. Furthermore, a volcano plot plots the effect sizes against the negative decimal logarithm (−log10) of the *p*-values. Additionally, using the multiple outcomes meta-analysis, a Venn diagram is produced to display how many genes were found to be statistically significantly over- or under-expressed in each condition. All the available plots are presented in Figure 3.

## 3. Results 

To illustrate the utility and features of the MAGE toolkit, we provide a simple real-life scenario (see the Supplementary Materials) where we analyzed ten published microarray case-control studies on placental samples [27]. These data were previously analyzed in a meta-analysis conducted by Vennou et al [27]. In the first step, for each study, the subject’s status of controls and cases was annotated. The datasets were sourced from different platforms, so we had to convert the probe identifiers to gene identifiers, and then combine the multiple entries of the same gene into one entry, using average value with the use of the GISU module. We performed univariate meta-analysis at an FDR level of 0.01. We also performed functional enrichment analysis using the differentially expressed genes (DEGs) derived from the meta-analysis. The functional enrichment analysis of the input gene list was performed using the g:Profiler toolkit. Each functional enriched term was derived from the most common data sources which are regularly updated (Gene Ontology, KEGG, Reactome, WikiPathways, miRTarBase, TRANSFAC, Human Protein Atlas, CORUM, and the Human Phenotype Ontology). The enrichment analysis results can be downloaded in Manhattan plot, heatmap, or table formats containing information about the functionally enriched term, gene associations, and corresponding *p*-values.

The results file contains information about the effect size, the standard error, the z-value, the *p*-value, the metrices for heterogeneity, and the statistical significance for each gene according to the *p*-value given by each correction method (Holmes, Bonferroni, FDR/Simes etc.) (Supplementary File S1, Figure S5). The effect size reveals which genes are overexpressed (the genes with a positive value) or underexpressed (the genes with a negative value). In our example, differentially expressed genes (DEGs) were considered to be those identified at a false discovery rate of 0.01, which is shown in the column “Simes”. The number 1 indicates the statistically significant DEGs, and 0 the non-statistically significant DEGs (the same for Holmes, Bonferroni, and the other correction methods). Meta-analysis identified 739 DEGs associated with preeclampsia. The molecular interactions among these DEGs, and the biochemical pathways in which these genes participate, were investigated with functional enrichment analysis. Finally, histograms were used to portray the measures of heterogeneity (Figure 3a), and a Q–Q plot, which shows the theoretical distribution of the corrected effect sizes compared against the actual distribution of the corrected effect sizes, is given (Figure 3d). In addition, the volcano plot is available; this depicts the effect sizes against the negative decimal logarithm (−log10) of the *p*-values (Figure 3b).

The enrichment results in g:GOSt are highlighted in a Manhattan plot (Figure 3e). The enrichment results are presented in a Manhattan Plot with all significant terms identified per source; this is accompanied by a more extensive readable output format with detailed information about every term, with a gene list and *p*-values. Each functional enriched term is derived from the most common data sources which are regularly updated such as Gene Ontology, KEGG, Reactome, WikiPathways, miRTarBase, TRANSFAC, Human Protein Atlas, CORUM, and the Human Phenotype Ontology. Furthermore, a heatmap visualization illustrates results for genes participating in significant enrichment terms.

More specifically, enrichment analysis returned DEGs that are highly overrepresented in terms from GO biological processes, molecular functions, cellular components, and regulatory motifs (GO:BP, GO:MF, GO:CC, TF). There were no significant results in functional terms from KEGG, Reactome, WikiPathways, miRTarBase, Human Protein Atlas, CORUM, or the Human Phenotype Ontology sources. The top five significant terms from each source are represented in Supplementary File S1, Table S1 with the corresponding heatmap visualization per source.

**Figure 4:**
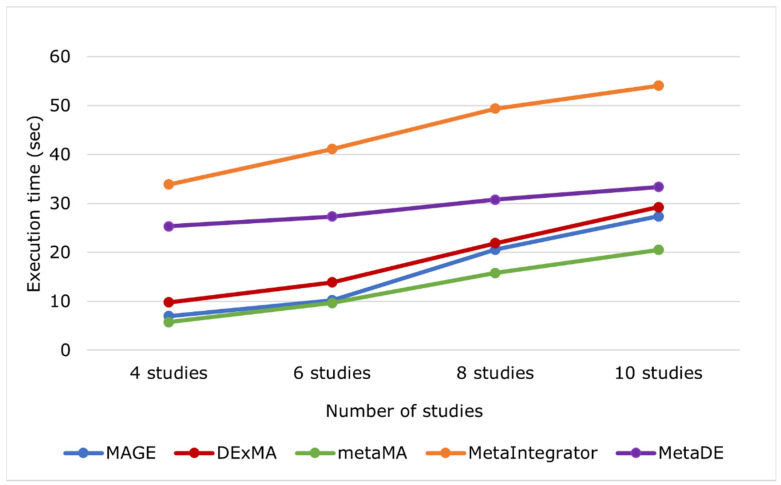
Run-time comparison of MAGE and other tools using different number of studies.

A comparison of MAGE against other relevant packages for the meta-analysis of gene expression data, such as metaMA, MetaIntegrator, DExMA, MetaDE, and Express Analyst, is provided in Table 1. In brief, MAGE implements the widest range of features, and it is, to our knowledge, the only publicly available implementation of bootstrap meta-analysis and multiple outcomes meta-analysis. Most of the tools mentioned in Table 1 incorporate only basic methods and need programming skills to configure and finally execute them (a process that requires excessive amount of work). In contrast to these other tools, MAGE is user-friendly, since it allows full parameterization through a configuration file, without requiring programming skills. Additionally, investigations into the time needed to perform an analysis, and the comparison against the other available tools (Table 2, Figure 4), show that MAGE is quite fast even with a large number of studies. metaMA is the only tool that slightly outperforms MAGE in terms of speed, but it offers significantly fewer options for meta-analysis. All packages provide functionalities for standard meta-analysis. 

**Figure 5:**
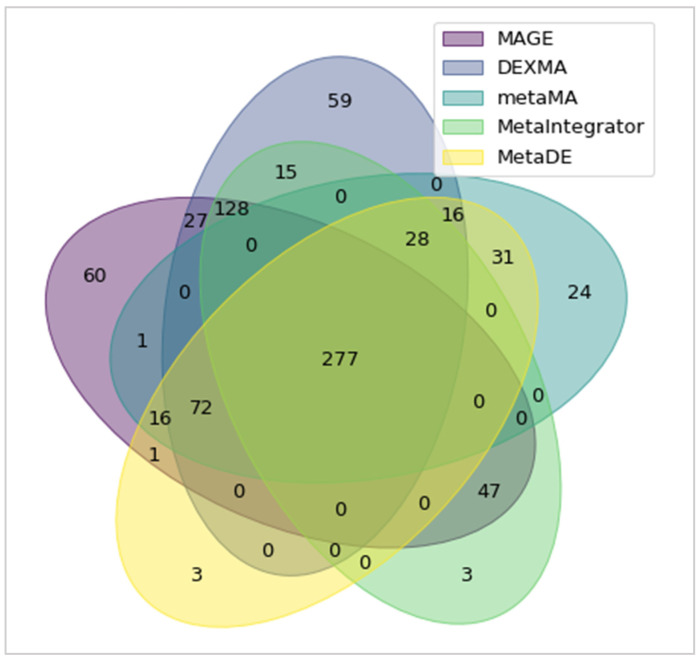
Venn diagram for the common genes that were identified as differentially expressed in each meta-analysis package.

For the comparison, we used the whole set described earlier, and subsets of the ten placental microarray case-control studies. Each study in this dataset contained twenty-five thousand genes on average. Vennou et al [27] performed this meta-analysis with STATA statistical software and identified 629 differentially expressed genes, at an FDR of 0.01. To compare the results between packages, we used the same meta-analysis settings. MAGE identified the same differentially expressed genes. Concerning the comparison with the other microarray meta-analysis packages, the results were similar when the same method was used, as expected. However, minor discrepancies may arise from the use of Cohen’s *d* or Hedge’s *g*. More specifically, DEXMA gave very similar results to MAGE, and identified 622 differentially expressed genes. The slight discrepancy can be explained by the fact that DEXMA uses the approximate correction for g, instead of the exact one. The other three tools identified significantly fewer differentially expressed genes. MetaDE (444 genes) and metaMA (446 genes) require that all studies must contain the same set of genes in order to perform the meta-analysis. This is not statistically necessary and may yield biased results (metaDE uses the Hedge’s *g* correction whereas metaMA does not). Finally, the MetaIntegrator package (498 genes) does not use the Hedge’s g correction at all and, for this reason, yields different results. The Venn diagram below (Figure 5) depicts the common genes that were identified as differentially expressed in each tool that was used in the comparison. A total of 277 differentially expressed genes were identified by all methods. For comparison purposes, all analyses were performed using a personal computer with the following technical characteristics: operating system (OS): Windows 10 Pro, CPU: Intel i7, 7th Gen. (7500U), 2.70 GHz and 2.90 GHz, RAM: 16 GB, and drive type: SSD.

## 4. Discussion

MAGE is a Python open-source software package for the meta-analysis of gene expression data. It includes useful features that can be used to detect differentially expressed genes in gene expression studies, and gives accurate estimations; at the same time, it is both easy to use and fast. For the first time, bootstrap meta-analysis and multiple outcomes meta-analysis are introduced as available functions in a package for the meta-analysis of gene expression; this may help researchers to produce more accurate results and address several research questions not easily addressed with other packages. MAGE is a complete package for gene expression meta-analysis, which only needs a set of gene expression studies as input to run all the available analyses, without the need for coding. Furthermore, the fact that MAGE is also available as a web service makes it a more user-friendly tool for users with limited knowledge of programming. 

One thing we need to bear in mind is that all meta-analysis tools and methods assume that the data are pre-processed and normalized in a proper manner [12,13]. However, batch effects are a source of significant problems in microarray data analysis, especially when it comes to combining different datasets. Although normalization methods do improve the situation, they cannot completely remove the effect, and hence dedicated methods are needed [28,29]. However, we should emphasize that microarray integration can be conceptually divided into approaches that perform early-stage integration (cross-platform normalization or data merging) and those that perform late-stage integration (meta-analysis) [30,31]. Since meta-analysis methods first perform a comparison within studies, removing batch effects becomes most important in the case of data merging (cross-platform normalization), and not in the traditional meta-analysis that we advocate in this work [30,31].

## 5. Conclusions

The MAGE toolkit is a web tool for the meta-analysis of gene expression studies. The package offers some unique features, such as the conversion of identifiers to gene symbols, and multiple outcomes meta-analysis, coupled with several useful tools for standard meta-analysis, graphical representation of results, functional enrichment, and ease of use. We have shown that MAGE is fast and accurate, and we contend that, due to its interactivity and ease-of-use, it is a useful tool for gene expression meta-analysis. It is designed for non-experts without prior familiarity with command line tools or scripting. Meta-analysis is the subject of active research in our lab, and thus MAGE will be continuously updated with new features (i.e., Bayesian meta-analysis, other random effects estimators, network meta-analysis, interactive plots, and more).

## Figures and Tables

**Table 1:** Comparison of features available in packages for the meta-analysis of gene expression.

Features	MAGE (2022)	metaMA (2009)	MetaDE (2012)	MetaIntegrator (2017)	Express Analyst (2019)	DExMA (2021)
Software type	Web based, Standalone	Standalone	Standalone	Standalone	Web based	Standalone
Programming language	Python	R	R	R	Javascript, R	R
License	Free	Free	Free	Free	Free	Free
Data Input	Expression tables	Expression tables	Expression tables	Expression tables	Expression tables	Expression tables
GEO data download	No	No	No	Yes	No	Yes
Probe annotation	Yes	No	Yes	Yes	Yes	No
Standard meta-analysis	Yes	Yes	Yes	Yes	Yes	Yes
Rank product meta-analysis	No	No	Yes	No	Yes	No
*p*-value combination	No	No	Yes	No	Yes	Yes
Hedge’s g	Yes	No	Yes	No	No	Yes
IDD/IRR	Yes	Yes	No	No	No	No
FDR methods	Yes	No	Yes	Yes	No	Yes
FWER methods	Yes	No	No	No	No	No
Bootstrap standard errors	Yes	No	No	No	No	No
Multiple outcomes meta-analysis	Yes	No	No	No	No	No
Enrichment analysis	Yes	No	Yes	No	Yes	No
Requires a common gene set across studies	No	Yes	Yes	No	No	No
Visualizations	Yes	Yes	Yes	Yes	Yes	Yes

**Table 2:** Evaluation of MAGE and other tools in terms of speed with varying number of studies.

Number of Studies	4 Studies	6 Studies	8 Studies	10 Studies
MAGE	6.98 s	10.23 s	20.58 s	27.36 s
DExMA	9.81 s	13.89 s	21.87 s	29.25 s
metaMA	5.74 s	9.67 s	15.81 s	20.54 s
MetaIntegrator	33.91 s	41.12 s	49.39 s	54.07 s
MetaDE	25.32 s	27.33 s	30.78 s	33.37 s

## Data Availability

Not applicable.

## Supplementary Materials

The following supporting information can be downloaded at: https://www.mdpi.com/article/10.3390/biology11060895/s1, Supplementary File S1: Set up tool parameters and Meta-analysis Results [27,32,33,34,35,36,37,38,39,40,41].
