# Supplementary material for: MAGE: An Open-Source Tool for Meta-Analysis of Gene Expression Studies
Source: Biology (Basel). 2022 Jun 10;11(6):895. doi: 10.3390/biology11060895 (PMC9220151; doi:10.3390/biology11060895)
Supplement: Supplementary file 1 [file biology-11-00895-s001.zip › biology-1746200-supplementary.pdf]

MAGE: a python package for Meta-analysis of gene expression studies

Ioannis A. Tamposis[#], Georgios A. Manios[#], Theodosia Charitou, Konstantina E. Vennou, Panagiota I. Kontou and Pantelis G. Bagos*

Department of Computer Science and Biomedical Informatics, University of Thessaly, 35100 Lamia, Greece

[#] These authors contributed equally

*To whom correspondence should be addressed.

Supplementary Material

Usage scenario

PART A – Set up tool parameters

Meta-analysis of gene expression data is widely used to identify differentially expressed genes that can be used as diagnostic and prognostic biomarkers of a disease. MAGE (Meta-Analysis of Gene Expression) can be used to perform a broad range of tasks related to meta-analysis of gene expression data. Figure S1 shows a schematic representation of the workflow, which is the example presented in this scenario.

A dataset of ten published microarray case-control studies on placental samples, regarding preeclampsia [1-10] was used. These data were previously analyzed in a meta-analysis conducted by Vennou et al [11]. In the first step each study file should be uploaded in tab-delimited format (.txt) as shown in the Figure S2. The first row should be named ID and the next columns contain the experiment subjects' name (e.g., GSMxxx). The second row should be named CLASS and the next columns contain subjects' status (for an analysis with a single outcome the CONTROL and the CASE was used to indicate the status of the controls and the patients of the study). The subsequent lines should have either the gene symbols identifiers of the experiment or the platforms probe identifiers (if the GISU module will be used) and the expression value of each probe per subject.

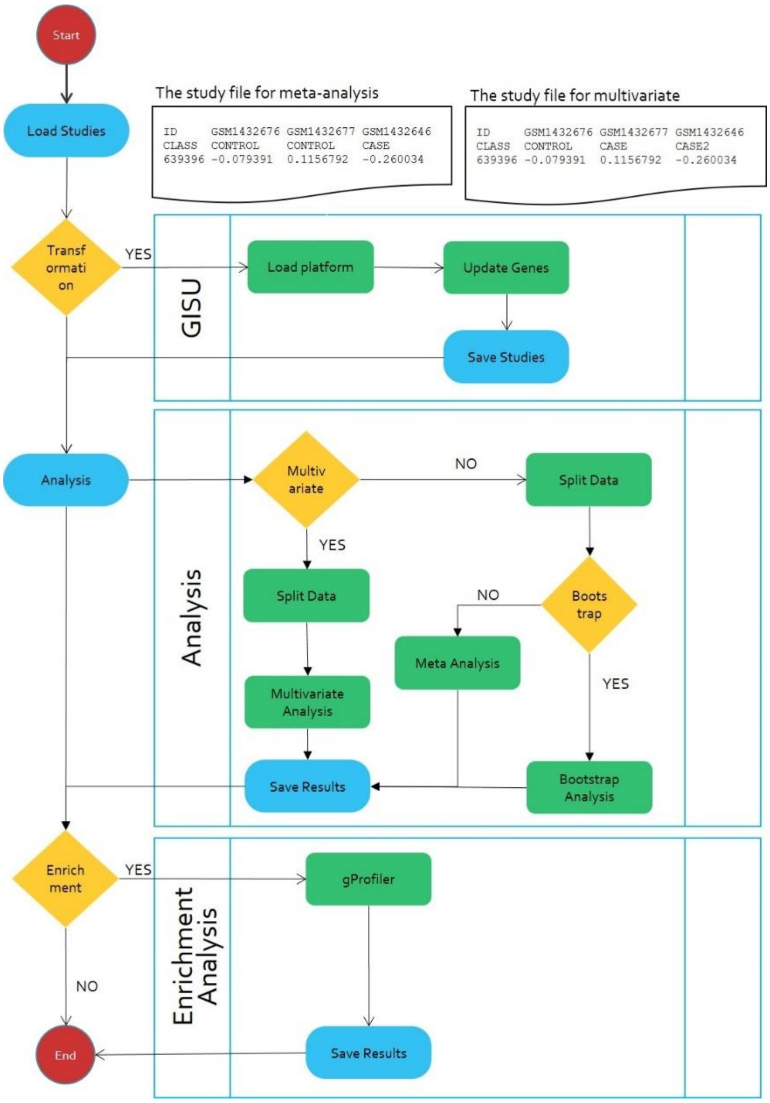

Figure S1. Schematic representation of the workflow

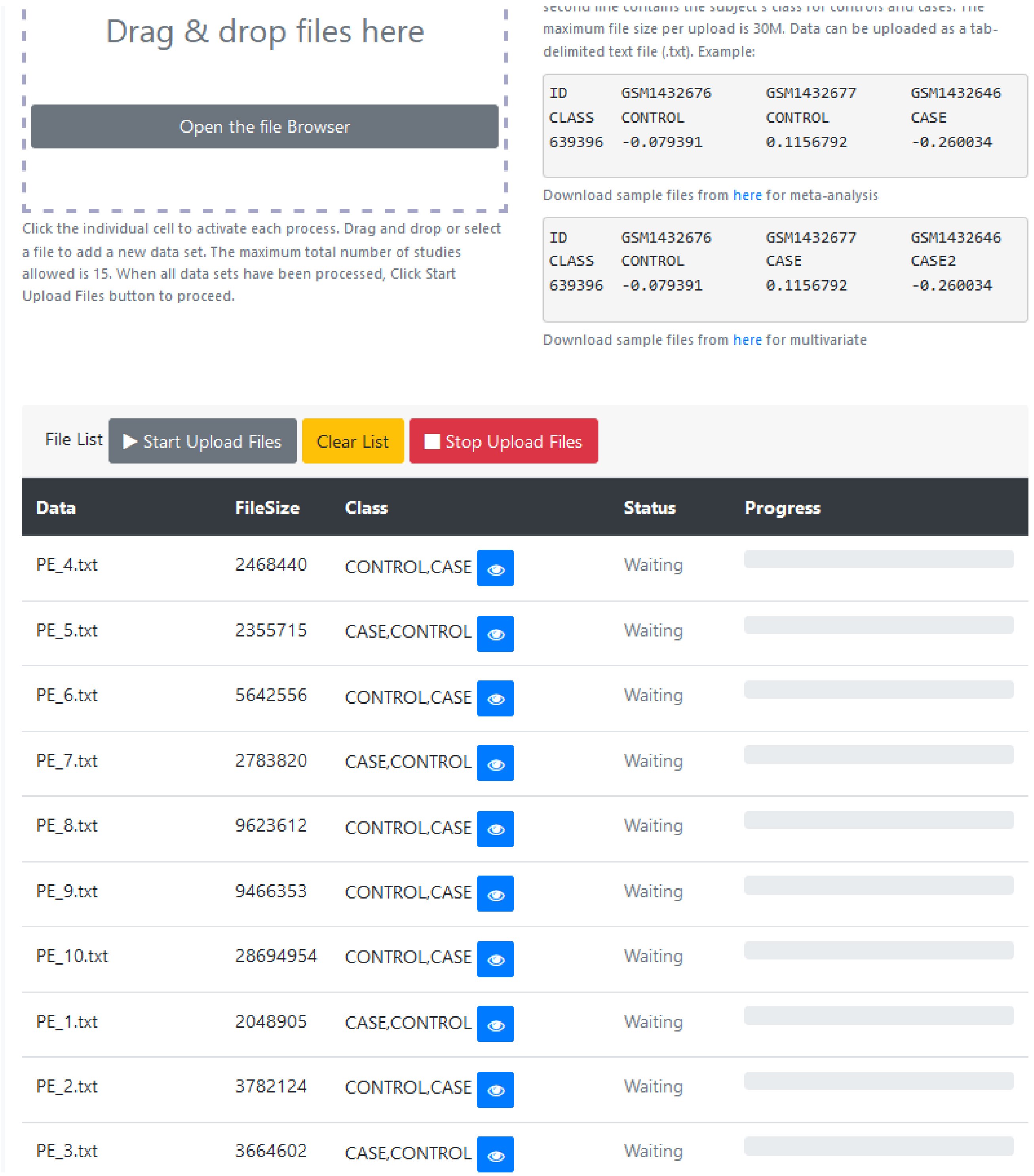

Figure S2. The web interface of the tool.

ID	GSM106249	GSM106250	GSM106274	GSM106275	GSM106276	GSM106277	GSM106278	GSM106279	GSM106280	GSM106281	GSM106282	GSM106283	GSM10628	GSM106285
CLASS	CONTROL	CONTROL	CONTROL	CONTROL	CASE	CASE	CASE	CASE	CASE	CASE	CASE	CASE	CASE	CASE
A1BG	0.07175	-0.00749	0.06815	0.063	0.00335	0.0205	0.00099	0.03792	0.0278	0.03205	-0.02005	0.03245	0.01948	0.05175
A1BG-AS1	-0.0637	0.182	-0.148	-0.176	-0.28	-0.174	-0.0894	-0.333	-0.232	-0.13	0.0734	-0.203	-0.0949	-0.215
A1CF	0.0927	0.117	-0.0838	-0.00781	-0.0689	-0.0132	-0.0774	-0.617	-0.11	0.0539	-0.0637	-0.0975	-0.132	-0.183
A2M	-0.00156	0.0791	0.0443	-0.0961	-0.251	-0.279	-0.15	-0.217	0.0465	-0.128	0.0422	-0.0206	-0.351	-0.206
A2ML1	0.0367	-0.0748	0.0633	0.138	0.104	0.039	0.0184	0.0108	0.262	-0.000297	-0.112	0.0158	0.0654	0.0512
A4GALT	-0.253	0.0246	-0.0369	0.0294	-0.205	-0.115	-0.142	-0.213	0.098	-0.195	-0.0864	-0.0518	0.0143	-0.106
A4GNT	0.0103	-0.174	-0.000934	0.168	0.216	0.0965	0.0852	0.115	0.107	-0.0177	-0.197	0.0167	-0.127	0.045
AAAS	-0.0785	-0.0139	-0.0564	-0.0718	-0.0997	-0.0892	-0.0491	-0.116	-0.0841	-0.0991	-0.039	-0.0849	-0.103	-0.0678
AACS	-0.0277	0.0988	-0.0469	-0.159	0.12	0.139	0.0441	0.0506	-0.0381	0.00619	0.14	-0.0544	0.0352	-0.0435
AADAC	-0.0393	-0.0871	0.163	-0.0743	-0.0301	0.0784	-0.0819	-0.0669	-0.0219	-0.201	-0.319	-0.0289	0.0576	-0.0164
AADACL2	0.0327	0.0431	0.0815	0.0727	0.0769	0.0991	0.092	0.088	0.0553	-0.0515	0.0652	0.0134	0.116	0.0225
AADAT	0.0761	0.0525	-0.079	0.199	0.0124	-0.0215	0.0822	0.104	0.0013	0.143	0.356	0.0865	0.237	0.0485
AAED1	0.234	0.109	-0.0757	0.0351	-0.118	0.032	0.128	0.231	0.125	0.166	0.455	0.0837	0.0457	0.052
AAGAB	0.0257	-0.0424	0.0194	0.0477	-0.0523	-0.0461	-0.0164	-0.0173	0.0449	-0.0287	-0.0484	-0.011	-0.0496	0.0324
AAK1	0.0042	-0.0449	-0.054615	0.037285	-0.05199	-0.0425	-0.07735	-0.07515	0.0055	0.0245	-0.08845	-0.001	0.0395	-0.01515
AAMDC	0.0272	0.191545	0.14435	0.1005	0.049705	-0.1145	-0.0314	-0.11945	-0.10465	0.0718	0.2945	0.05085	0.0336	0.11179
AAMP	-0.3	0.0348	-0.0539	-0.164	-0.135	0.101	-0.0386	-0.022	-0.138	0.0379	0.216	-0.011	-0.0731	-0.0533
AANAT	0.0101	0.0243	-0.0483	-0.0311	-0.0408	0.0787	-0.00321	-0.0423	-0.074	0.0147	-0.0516	-0.00112	-0.121	0.0248
AAR2	-0.0205	0.0112	-0.000208	-0.0299	0.00483	0.00299	-0.00266	-0.0296	-0.0358	0.0081	-0.0772	0.0296	-0.0353	-0.0188
AARS	-0.119	0.129	-0.0697	-0.0955	-0.0713	0.0496	-0.0377	-0.0699	-0.171	0.107	0.3	-0.0578	-0.117	-0.0339
AARS2	0.017	0.0391	0.045	-0.0433	-0.0247	0.0203	-0.0152	-0.0218	-0.0337	-0.0276	-0.0519	-0.0219	-0.00352	-0.0678
AARSD1	-0.0195	-0.057	-0.1092	-0.3725	0.0485	0.13325	0.10925	0.0787	0.13095	-0.014665	0.05875	0.0682	0.1515	0.107
AASDH	0.027233	-0.008567	-0.020667	-0.059267	-0.0854	-0.02298	-0.007567	0.0454	-0.047333	-0.079867	0.4059	-0.071033	-0.027533	-0.11923
AASDHPPT	-0.077483	0.0027	0.050263	-0.113967	-0.011233	0.023933	0.039433	0.0971	0.03516	0.024867	0.118133	0.054933	0.019533	0.00283
AASS	0.0282	0.159	-0.0444	0.0921	0.0725	0.0339	-0.0384	0.054	0.261	0.0368	0.132	-0.0488	-0.0812	-0.0819
AATF	-0.194	0.05195	-0.0706	-0.1425	0.1216	0.1615	0.1068	0.06875	-0.10655	0.05325	0.1305	0.01652	-0.05145	-0.006454
AATK	-0.0705	0.038533	0.05957	0.09471	-0.109333	-0.076233	-0.0568	-0.0691	0.149567	-0.074787	-0.120333	0.056393	0.084	-0.028967
ABAT	0.10305	0.11255	0.115	0.054	-0.0238	0.05977	-0.0672	0.02175	-0.17005	-0.10194	0.1155	-0.05565	-0.06225	0.01435
ABCA1	-0.024275	0.054984	-0.054917	-0.0363	0.028134	0.039584	0.015833	0.021916	-0.008323	-0.037267	0.010834	0.00225	0.062445	-0.061217
ABCA10	-0.0837	-0.106	0.141	-0.198	-0.144	-0.274	-0.163	-0.0815	-0.0472	-0.164	-0.137	-0.0601	0.00143	-0.0378
ABCA11P	-0.0758	-0.119	0.183	-0.173	-0.00636	-0.155	-0.0257	-0.0229	0.0465	-0.0909	-0.0751	-0.187	-0.0898	-0.112
ABCA12	-0.0942	-0.118	0.226	0	0.463	0.432	-0.0696	0.288	0.108	-0.0522	0.00989	0.127	0.514	0.305
ABCA13	0.0587	-0.09	0.0208	-0.349	-0.0442	0.35	-0.0435	0.0918	0.0469	0.077	0.000374	0.315	0.121	0.6
ABCA2	-0.0597	0.0314	-0.04	0.0327	-0.175	-0.121	-0.175	-0.24	-0.126	-0.0471	-0.112	-0.0206	-0.0437	-0.094
ABCA3	0.0978	0.16	0.0252	0.0286	-0.188	-0.0438	-0.0528	-0.117	0.00623	0.0519	-0.0414	-0.0267	-0.386	-0.0792
ABCA4	0.0202	-0.246	-0.0184	0.239	-0.105	-0.233	0.0339	-0.0801	0.072	0.116	0.0335	0.145	0.142	0.39
ABCA5	0.0775	0.005	0.128	0.1142	0.1695	-0.122	-0.073	-0.028595	-0.12215	-0.147	0.0205	-0.04865	-0.09465	0.03875
ABCA6	0.226	0.195	0.0509	0.126	0.211	0.172	-0.00389	0.00198	-0.04	0.28	0.379	0.0827	-0.0705	-0.0206
ABCA7	-0.109	0.0552	0.166	0.166	0.0523	0.0544	0.0849	-0.11	0.286	0.0313	-0.22	0.0612	0.201	0.0898
ABCA8	0.0772	0.0444	0.219	0.304	0.258	0.17	0.0342	-0.0121	-0.00143	0.144	0.075	0.31	0.266	0.193
ABCA9	0.05	-0.422	-0.115	-0.0595	0.458	0	0.436	0.196	0.1	0.503	0.616	-0.105	-0.356	-0.166
ABCB1	-0.000332	0.0627	-0.0802	0.0526	0.0254	0.415	0.00329	0.28	-0.139	0.0603	0.263	-0.0402	-0.129	0.139
ABCB10	0.01615	0.10258	-0.01418	0.023825	0.05975	-0.019048	0.066625	0.134875	0.029975	0.073243	0.13575	0.1252	0.03935	0.080025
ABCB11	0.00442	-0.183	0.00843	-0.0121	0.000151	-0.0763	-0.0315	-0.0426	-0.0803	-0.0431	-0.076	-0.0118	0.0275	0.0235
ABCB4	0.165	0.0807	-0.0245	0.0699	0.136	0.369	0.0143	0.249	-0.0277	0.366	0.127	0.288	0.142	0.151
ABCB5	0.103	-0.0849	0.00755	0	0.153	-0.0717	-0.127	-0.296	-0.196	0.406	0.174	0.149	0.208	0.135
ABCB6	-0.03845	0.02485	-0.06415	-0.032455	-0.09485	-0.205	-0.0668	-0.2565	-0.0807	-0.035775	-0.08145	-0.018882	-0.060819	0.017905
ABCB7	0.0859	0.0264	0.0885	0.0156	0.0143	-0.0348	-0.00159	0.0283	0.0545	0.092	0.184	0.112	-0.0749	0.0301
ABCB8	1.37	-0.256	-0.0386	-0.0826	-0.181	-0.117	0.0142	-0.089	-0.198	-0.0584	0.617	-0.219	-0.0226	-0.157
ABCB9	0.05825	0.1435	0.10985	0.1117	0.04805	0.1181	0.02285	0.06063	0.1272	0.0667	-0.00975	0.05045	0.05935	0.03134
ABCC1	-0.0401	0.00802	0.476	0.187	-0.348	-0.159	-0.0676	-0.139	-0.0545	0.0618	-0.0608	0.387	0.215	0.245
ABCC10	-0.0144	-0.144	-0.0252	-0.0355	-0.0309	-0.0422	0.000469	-0.0303	-0.0456	0.0185	-0.0162	-0.0495	0.0489	-0.0655
ABCC11	0.0832	-0.116	0.0509	-0.131	0.029	0.254	-0.099	0.0229	-0.161	-0.0272	0.361	0.0485	-0.12	0.09
ABCC12	0.539	-0.214	0.154	0	-0.403	-0.0595	-0.00643	0	-0.0589	-0.316	-0.254	0.0269	0	0.373
ABCC13	-0.027953	0.024	-0.016253	0.03	0.047767	-0.0203	0.022	0.0463	-0.032443	-0.014333	-0.002167	-0.000767	-0.089633	0.025474
ABCC2	0.04045	0.1804	0.05535	0.0021	-0.034915	-0.017065	-0.02148	-0.0455	-0.02481	0.10355	0.03185	-0.0367	-0.00805	-0.12715
ABCC3	0.102	-0.177	0.022	-0.0529	-0.254	-0.201	0.0978	-0.0782	-0.242	0.0127	0.0382	-0.163	-0.0861	-0.0242
ABCC4	-0.225333	-0.417667	0.0972	-0.05	-0.124	-0.2021	-0.114533	-0.081	-0.208433	-0.364667	-0.405	0.1274	0.036	0.167367

Figure S3. Study file example

The next step is to define the tool parameters (Figure S4). While setting up the parameters, the user may choose the type of meta-analysis. The univariate meta-analysis is the default type. For multivariate meta-analysis, the user may select YES in the multivariate meta-analysis option. Moreover, the user may choose the level of significance from the drop-down menu, select the multiple comparison correction methods, and perform functional enrichment analysis. When all parameters were set, the user should click the execute button (Optionally the user may enter an email address to get the results). In this scenario, we conducted univariate meta-analysis with 0.01 level of significance, selecting all the multiple comparison correction methods, and performing functional enrichment analysis using the Benjamini – Hochberg FDR significance threshold.

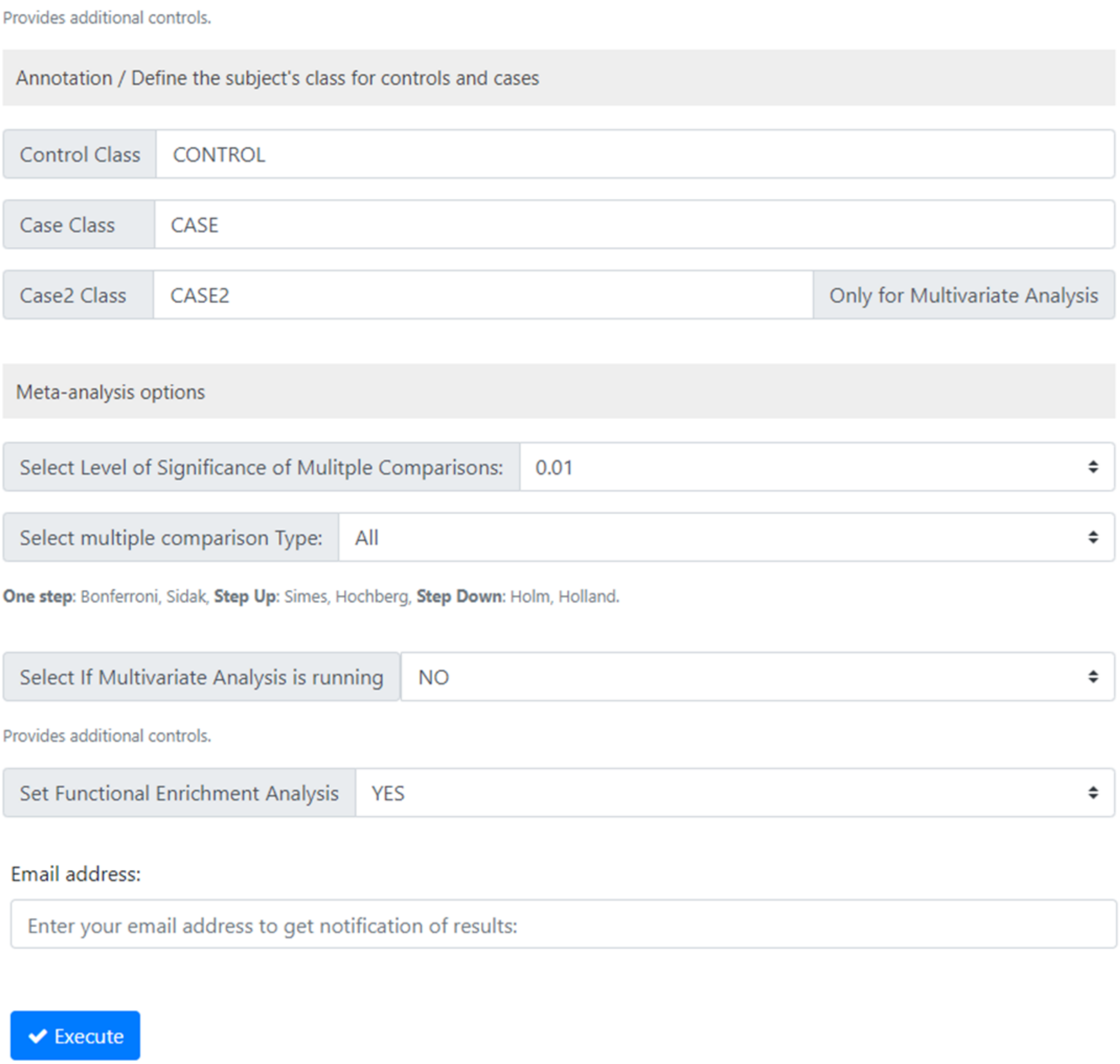

Figure S4. Web Interface tool parameters.

PART B – Meta-analysis Results

The results file contains information about the Effect size, the Standard Error, the *z*-value, the *p*-value, the metrices for heterogeneity and the statistical significance of each gene according to the *p*-value of each correction method (Holmes, Bonferroni, FDR/Simes etc.) (Figure 5). The Effect size reveals which genes are overexpressed (the genes with a positive value) or underexpressed (the genes with negative value). In our example, differentially expressed genes (DEGs) were considered those identified at a False Discovery Rate of 0.01, which is shown in the column "Simes". The number 1 indicates the statistically significant DEGs and the 0 the non-statistically significant DEGs (the same for Holmes, Bonferroni, and the other correction methods). Meta-analysis identified 739 DEGs associated with preeclampsia. The molecular interactions among these DEGs and the biochemical pathways in which these genes participate were investigated with functional enrichment analysis. Finally, histograms were used to portray the measures of heterogeneity (Figure 6) and qqplot that shows the theoretical distribution of the corrected effect sizes compared against the actual

distribution of the corrected effect sizes (Figure S7). Also, the volcano plot is available depicting the effect sizes against the negative decimal logarithm (-log10) of the *p*-values (Figure S8).

Genes	Effect size (Hedge's g)	Standard_Error	Q	I_Squared	Tau_Squared	p_Q_value	z_test_value	p_value	bonferroni	sidak	holm	holland	hochberg	simes
A1BG	-0.187483812	0.124675674	10.65026	24.88	0.03300505	0.225	1.503772199	0.139	0	0	0	0	0	0
A1BG_AS1	-0.464371052	0.23264274	0.790917	0	0	0.851	1.996069387	0.046	0	0	0	0	0	0
A1CF	-0.05634002	0.175202881	23.94549	62.41	0.17268501	0.004	0.321570169	0.748	0	0	0	0	0	0
A2M	-0.361338851	0.17602856	23.95134	62.42	0.17440305	0.004	2.052728544	0.04	0	0	0	0	0	1
A2ML1	0.100899543	0.13155626	11.64866	31.32	0.04536663	0.173	0.766968765	0.445	0	0	0	0	0	0
A4GALT	0.071338351	0.125804837	12.96911	30.6	0.04501624	0.17	0.567055706	0.572	0	0	0	0	0	0
A4GNT	0.150358024	0.103690355	8.481419	5.68	0.00582605	0.389	1.450067596	0.154	0	0	0	0	0	0
AAAS	-0.182881964	0.201491671	31.56084	71.48	0.26466705	0	0.907640315	0.366	0	0	0	0	0	0
AACS	0.054171429	0.097786	5.796878	0	0	0.67	0.553979391	0.58	0	0	0	0	0	0
AADAC	0.087611513	0.158216439	16.68008	52.04	0.106149	0.034	0.553744688	0.58	0	0	0	0	0	0
AADACL2	0.212328147	0.109663242	6.10259	1.68	0.00163949	0.413	1.936183376	0.053	0	0	0	0	0	0
AADAT	0.081504594	0.258347902	35.8873	80.49	0.39974553	0	0.315483861	0.753	0	0	0	0	0	0
AAED1	-0.181036976	0.273535618	28.4666	78.92	0.3765811	0	0.661840594	0.508	0	0	0	0	0	0
AAGAB	0.078658842	0.151484908	15.3819	47.99	0.08993425	0.052	0.519252005	0.604	0	0	0	0	0	1
AAK1	0.041828521	0.188381777	17.9377	60.98	0.15629099	0.013	0.222041227	0.825	0	0	0	0	0	1
AAMDC	-0.312541845	0.107029493	8.734696	8.41	0.00902262	0.367	2.920146927	0.004	0	0	0	0	0	0
AAMP	-0.094961823	0.23049829	34.84092	77.04	0.33760929	0	0.411984936	0.681	0	0	0	0	0	0
AANAT	0.254716422	0.171089418	19.18733	58.31	0.13899459	0.015	1.488791213	0.143	0	0	0	0	0	0
AAR2	-0.174780083	0.132059733	11.99427	33.3	0.04860457	0.159	1.323492626	0.188	0	0	0	0	0	0
AARS	0.073040255	0.166558083	18.3289	56.35	0.12694764	0.019	0.438527233	0.662	0	0	0	0	0	0
AARS2	-0.025722358	0.101091297	6.857593	0	0	0.445	0.254446805	0.799	0	0	0	0	0	1
AARSD1	0.210028491	0.264434632	20.88218	71.27	0.32733657	0.002	0.794254861	0.428	0	0	0	0	0	0
AASDH	-0.155280198	0.154850122	12.90514	45.76	0.07965925	0.075	1.002777369	0.317	0	0	0	0	0	0
AASDHPPT	-0.257812739	0.243648023	37.86854	78.87	0.38453311	0	1.058135978	0.291	0	0	0	0	0	0
AASS	-0.020126664	0.099814004	8.154321	1.89	0.0018639	0.419	0.201641682	0.84	0	0	0	0	0	0

Figure S5. **Meta-analysis results file**. This table provides detailed information about the results from meta-analysis for each gene: the effect size, the standard error, the metrics for heterogeneity (*Q*, *I* squared and Tau Squared), the *p*-value for heterogeneity (*p*-*q*-value), the *z* test value, the *p*-value, and the results per method of corrected p-values.

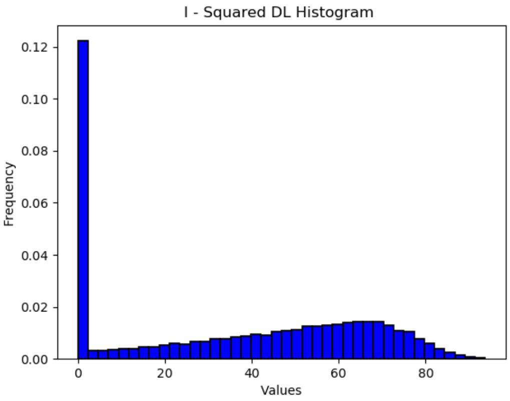

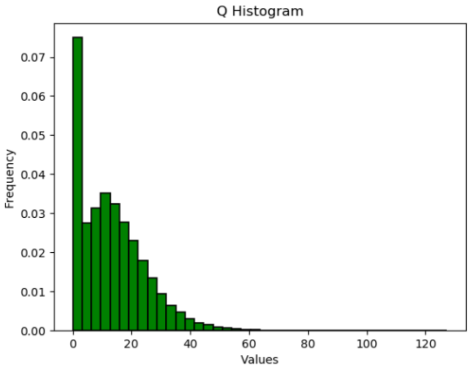

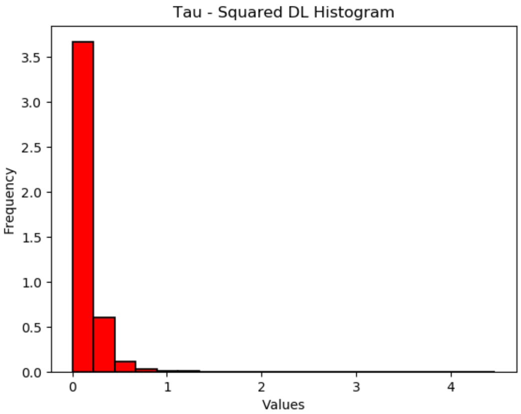

Figure S6. Heterogeneity histograms for *I*-Squared, Tau-Squared and *Q* as shown in the meta-analysis results.

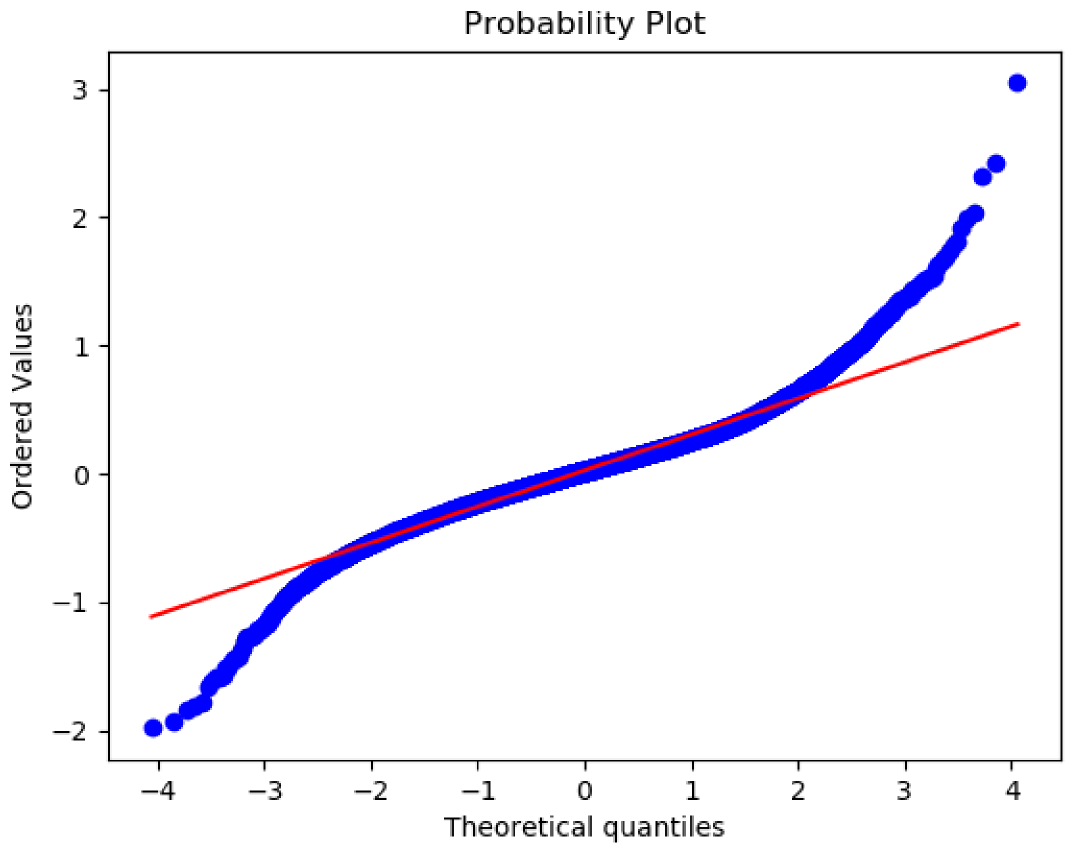

Figure S7. QQ plot

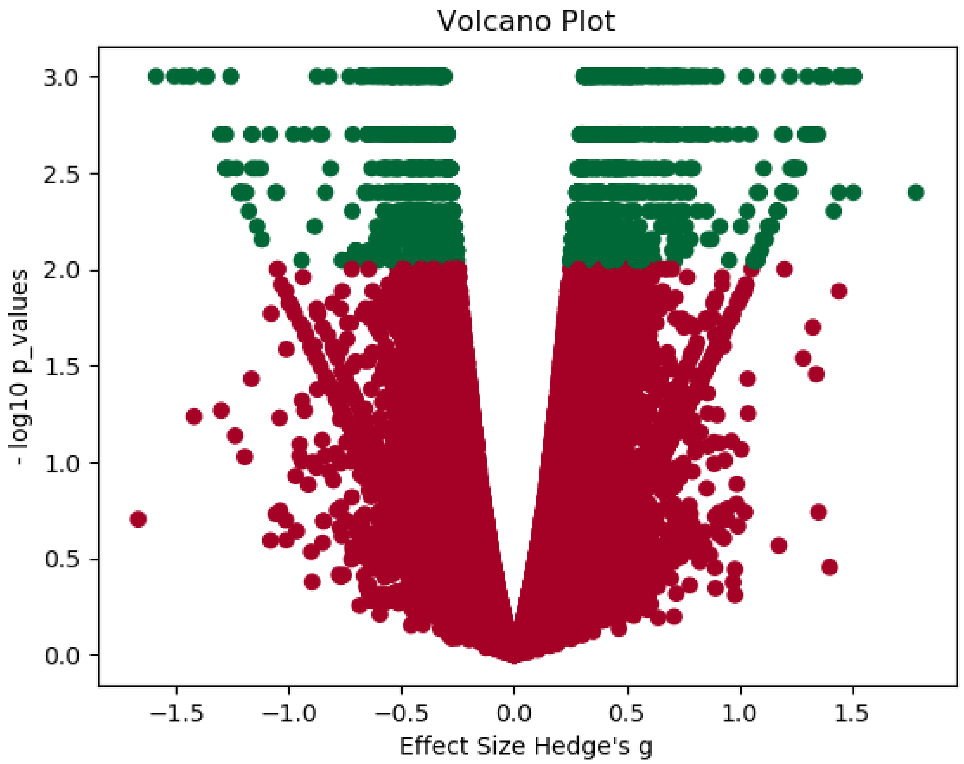

Figure S8. Volcano plot

PART C - Functional Enrichment Analysis

We also performed functional enrichment analysis, also known as over-representation analysis (ORA), or gene set enrichment analysis, using the differentially expressed genes (DEGs) derived from the meta-analysis. The functional enrichment analysis of the input gene list is performed using the g:Profiler toolkit in python using the g:GOSt core tool that detects statistically significantly enriched biological processes, molecular functions, cellular components, biological pathways, regulatory motifs and protein complexes using a hypergeometric test with default parameters (alpha = 0.01, enrichment_analysis = YES, organism = hsapiens, threshold = 0.05, threshold_method=fdr).

The enrichment results in g:GOSt are highlighted in a Manhattan plot (Figure S9). The enrichment results are presented in a Manhattan Plot with all significant terms identified per source and it is accompanied by a more extensive readable output format with detailed information about every term with gene list and p-values. Each functional enriched term is derived from the most common data sources which are regularly updated such as Gene Ontology, KEGG, Reactome, WikiPathways, miRTarBase, TRANSFAC, Human Protein Atlas, CORUM, and the Human Phenotype Ontology. Furthermore, a heatmap visualization illustrates results for genes participating in significant enrichment terms (Figure S10).

In more detail, enrichment analysis returned DEGs that are highly overrepresented in terms in GO biological processes, molecular functions, cellular components and regulatory motifs (GO:BP, GO:MF, GO:CC, TF). There weren’t any significant results in functional terms from KEGG, Reactome, WikiPathways, miRTarBase, Human Protein Atlas, CORUM, and the Human Phenotype Ontology sources. The top 5 significant terms at each source are represented in Table S1 with the corresponding heatmap visualization per source in Figure S10.

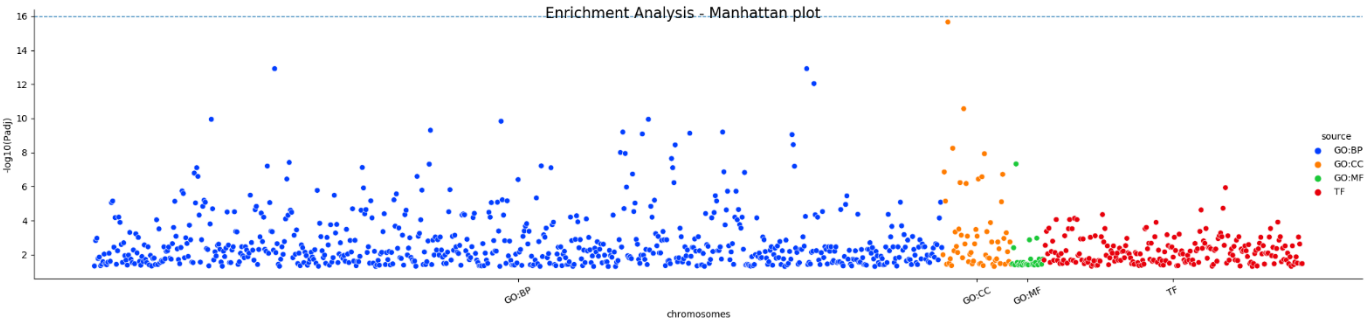

Figure S9. **Manhattan Plot**. This plot maps genes to known functional information sources and detects statistically significant enriched terms using the well-proven cumulative hypergeometric test. The x-axis shows the functional terms, and the y-axis shows the corresponding enrichment p-values in negative log10 scale. Each circle represents the significant functional term across all the analysed term categories. The circles are colour-grouped by data source.

Table S1. Functional enrichment output. The first 5 significant terms are shown for Gene Ontology (Biological Processes (BP), Molecular Function (MF), Cellular Component (CC) and Transcription Factor Regulatory Motifs source with term name, ID and adjusted p-values.

source	term_name	term_id	adjusted_p_value	negative_log10_of_adjusted_p_value
GO:BP	response to organic substance	GO:0010033	5.54E-07	6.256117189
GO:BP	cellular response to chemical stimulus	GO:0070887	1.25257E-06	5.902196824
GO:BP	response to chemical	GO:0042221	4.66537E-06	5.331113705
GO:BP	cellular response to organic substance	GO:0071310	1.01484E-05	4.993600908
GO:BP	multicellular organism development	GO:0007275	0.002198326	2.657907808
GO:CC	vesicle	GO:0031982	3.24914E-05	4.488232185
GO:CC	cytoplasm	GO:0005737	5.69367E-05	4.244607716

GO:CC	endomembrane system	GO:0012505	0.000145738	3.83642802
GO:CC	cytoplasmic vesicle	GO:0031410	0.000193625	3.713038346
GO:CC	integral component of plasma membrane	GO:0005887	0.000193625	3.713038346
GO:MF	monosaccharide binding	GO:0048029	0.004247765	2.371839509
GO:MF	peptidyl-proline dioxygenase activity	GO:0031543	0.019347834	1.713367656
GO:MF	L-ascorbic acid binding	GO:0031418	0.019347834	1.713367656
GO:MF	procollagen-proline dioxygenase activity	GO:0019798	0.023866897	1.622204046
GO:MF	peptidyl-proline 4-dioxygenase activity	GO:0031545	0.033468303	1.475366311
TF	Factor: MAZ; motif: GGGMGGGGSSGGGGGGGGGGG; match class: 1	TF:M09636_1	9.57247E-05	4.018976204
TF	Factor: PATZ; motif: GGGGNGGGGGMKGGRRNGGNRN	TF:M10026	0.000143196	3.844068332
TF	Factor: MAZ; motif: GGGMGGGGSSGGGGGGGGGGG	TF:M09636	0.000936755	3.028373962
TF	Factor: MAZ; motif: GGGGGAGGGGGNGRGRRRGNRG	TF:M09984	0.000936755	3.028373962
TF	Factor: GKLF; motif: NNNRGGNGNGGSN; match class: 1	TF:M07289_1	0.001458223	2.836175963

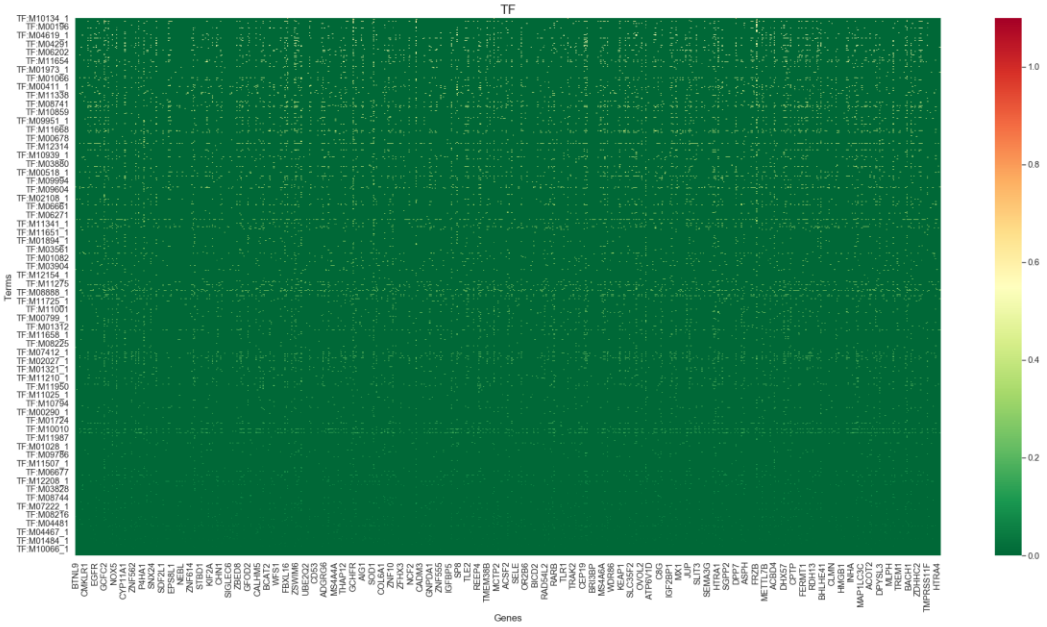

Figure S10. Heatmap plot of regulatory motifs in DNA according to putative transcription factor binding sites (TFBSs) from TRANSFAC database (TF). In the x-axis there are genes enriched for terms of TF in y-axis.

References

1. Guo, L., et al., *Differentially expressed microRNAs and affected biological pathways revealed by modulated modularity clustering (MMC) analysis of human preeclamptic and IUGR placentas.* Placenta, 2013. **34**(7): p. 599-605.
2. Jebbink, J.M., et al., *Increased glucocerebrosidase expression and activity in preeclamptic placenta.* Placenta, 2015. **36**(2): p. 160-9.
3. Leavey, K., et al., *Unsupervised Placental Gene Expression Profiling Identifies Clinically Relevant Subclasses of Human Preeclampsia.* Hypertension, 2016. **68**(1): p. 137-47.
4. Liang, M., et al., *Gene expression profiling reveals different molecular patterns in G-protein coupled receptor signaling pathways between early- and late-onset preeclampsia.* Placenta, 2016. **40**: p. 52-9.
5. Meng, T., et al., *Identification of differential gene expression profiles in placentas from preeclamptic pregnancies versus normal pregnancies by DNA microarrays.* OMICS, 2012. **16**(6): p. 301-11.
6. Nishizawa, H., et al., *Comparative gene expression profiling of placentas from patients with severe pre-eclampsia and unexplained fetal growth restriction.* Reprod Biol Endocrinol, 2011. **9**: p. 107.
7. Nishizawa, H., et al., *Microarray analysis of differentially expressed fetal genes in placental tissue derived from early and late onset severe pre-eclampsia.* Placenta, 2007. **28**(5-6): p. 487-97.
8. Sitras, V., et al., *Differential placental gene expression in severe preeclampsia.* Placenta, 2009. **30**(5): p. 424-33.
9. Tsai, S., et al., *Transcriptional profiling of human placentas from pregnancies complicated by preeclampsia reveals disregulation of sialic acid acetylesterase and immune signalling pathways.* Placenta, 2011. **32**(2): p. 175-82.
10. Winn, V.D., et al., *Severe preeclampsia-related changes in gene expression at the maternal-fetal interface include sialic acid-binding immunoglobulin-like lectin-6 and pappalysin-2.* Endocrinology, 2009. **150**(1): p. 452-62.
11. Vennou, K.E., et al., *Meta-analysis of gene expression profiles in preeclampsia.* Pregnancy Hypertens, 2020. **19**: p. 52-60.
